# Stress Management: How the Endoplasmic Reticulum Mitigates Protein Misfolding and Oxidative Stress by the Dual Role of Glutathione Peroxidase 8

**DOI:** 10.3390/biom15060847

**Published:** 2025-06-10

**Authors:** Yong Yang, Hao Peng, Danni Meng, Zizhu Fa, Chen Yao, Xinyu Lin, Joel Schick, Xiang Jin

**Affiliations:** 1 Ministry of Education Key Laboratory for Ecology of Tropical Islands, Key Laboratory of Tropical Animal and Plant Ecology of Hainan Province, College of Life Sciences, Hainan Normal University, Haikou 571158, China; yangyong@hainnu.edu.cn (Y.Y.); dannimeng@hainnu.edu.cn (D.M.); fazizhu130@hainnu.edu.cn (Z.F.); yaochen426@hainnu.edu.cn (C.Y.); xinyulinshiguang823@hainnu.edu.cn (X.L.); 2 Dongzhaigang Mangrove Wetland Ecosystem Hainan Observation and Research Station, Haikou 571158, China; 3 Genetics and Cellular Engineering Group, Research Unit Signaling and Translation, Helmholtz Zentrum Munich, Ingolstaedter Landstr. 1, 85764 Neuherberg, Germany; hao.peng@helmholtz-munich.de

**Keywords:** endoplasmic reticulum, oxidative stress, reactive oxygen species, unfolded protein response, GPX8

## Abstract

The endoplasmic reticulum mediates essential processes such as protein folding, transport, and post-translational modifications. Disruptions in endoplasmic reticulum function can lead to the accumulation of unfolded or misfolded proteins, initiating endoplasmic reticulum stress. This stress activates the unfolded protein response, a multifaceted signaling pathway aimed at restoring proteostasis, which is crucial for cellular survival and fate determination. This review summarizes the current knowledge of three major branches of the unfolded protein response: the IRE1, PERK, and ATF6 signaling pathways. A key novel component in endoplasmic reticulum stress adaptation is the redox-sensitive enzyme glutathione peroxidase 8 (GPX8), which plays a dual role in detoxifying hydrogen peroxide and supporting proper protein folding. By connecting unfolded protein response branches, GPX8 reduces oxidative damage while maintaining redox homeostasis, emphasizing its importance in endoplasmic reticulum stability. Furthermore, plant glutathione peroxidases exhibit parallel functions in endoplasmic reticulum redox homeostasis and unfolded protein response activation, highlighting the evolutionary conservation of this protective mechanism across kingdoms. Understanding the intricate relationship between GPX8, endoplasmic reticulum stress, and unfolded protein response signaling provides novel insights into therapeutic strategies for diseases characterized by protein folding defects and oxidative stress.

## 1. Introduction

Redox balance, the equilibrium between oxidation and reduction reactions, is crucial in various biological, chemical, and pathological processes. Disruptions of redox balance lead to the oxidative damage of biomolecules such as proteins, lipids, and DNA, resulting in disease or death [1]. Reactive oxygen species (ROS) play an important role in biological systems, significantly impacting redox balance. However, the role ROS play in biological processes could be dual.

On one hand, common ROS, for instance hydrogen peroxide (H_2_O_2_), can regulate signaling cascades such as the mitogen-activated protein kinase (MAPK) pathway, influencing cell proliferation and differentiation [2]. On the other hand, ROS overproduction can initiate oxidative stress, a condition characterized by a difference between ROS generation and antioxidant defenses [3]. Oxidative stress occurs from unmitigated highly reactive molecules derived from oxygen, including superoxide (O_2_•^−^), hydrogen peroxide (H_2_O_2_), hydroxyl radicals (•OH), and others, which play dual roles in cellular signaling and oxidative stress. Under conditions of oxidative stress, the sulfhydryl (-SH) groups of cysteine residues in proteins can undergo progressive oxidation to sulfenic (-SOH), sulfinic (-SO_2_H), and sulfonic (-SO_3_H) acids. Equally significant is the oxidative breakdown of existing disulfide bonds in folded proteins. These oxidative modifications have profound consequences for protein structure and function: they not only disrupt proper protein folding by interfering with the formation of native disulfide bonds, but also cause denaturation of already folded proteins through both conformational destabilization and disulfide bond cleavage. Together, these reactions represent fundamental molecular mechanisms by which reactive oxygen species impair proteostasis and contribute to cellular dysfunction. While low levels of ROS are essential for physiological processes like immune response and cell signaling, excessive ROS production can damage biomolecules and contribute to diseases such as cancer, neurodegeneration, and cardiovascular disorders. To compensate for the levels of ROS and alleviate oxidative stress, mammals have evolved a sophisticated ROS scavenging system. This system comprises various enzymes that play crucial roles in detoxifying ROS and maintaining redox balance, such as catalase (CAT), thioredoxin reductases (TXNRDs), superoxide dismutase (SOD), and glutathione peroxidases (GPXs) [4,5].

This review focuses on the sensitive relationship between redox homeostasis and endoplasmic reticulum (ER) function, particularly in the context of oxidative stress and protein folding. To gain insights into the mechanisms by which cells maintain ER stability and moderate oxidative damage, this review focuses on the protein GPX8, which serves as a nexus between protein (re-)folding and oxidative stress, balancing misfolded proteins through H_2_O_2_ detoxification. GPX8’s role in scavenging H_2_O_2_ within the ER not only protects against oxidative damage but also supports the proper folding of proteins, highlighting its critical function in maintaining cellular homeostasis under stress conditions.

## 2. ER Stress Response

The ER is a dominant organelle in the secretory pathway, responsible for protein folding, protein transport, and post-translational modifications. Alterations in ER function can lead to the accumulation of unfolded or misfolded proteins, a condition known as ER stress. The process of ER stress triggers the unfolded protein response (UPR), which comprises a series of precisely composed intracellular signaling reactions aimed at reducing the burden of unfolded proteins, thereby maintaining cellular vitality and function, which will highly relate to following cell fate [6]. The essential role of the ER is tightly linked to its stress responses, which in turn exhibit a relationship between protein folding and reactive oxygen species protection and mitigation.

### 2.1. UPR

The UPR is mediated by three signaling proteins: IRE1α (inositol-requiring enzyme 1α), PERK (protein kinase RNA-like ER kinase), and ATF6 (activating transcription factor 6). These key factors regulate adaptive processes through both transcriptional and non-transcriptional responses, impacting nearly all aspects of the secretory pathway, including protein folding, ER-associated degradation (ERAD), protein entry into the ER, secretion, and cell death [6,7]. Recent advances indicate that the UPR pathway plays significant roles in various physiological processes that are not directly related to protein folding as well. For example, the UPR interacts with critical signaling pathways that regulate lipid and energy metabolism, programmed cell death, immune responses, and cellular differentiation [8,9,10,11,12,13].

### 2.2. The Role of the IRE1 Pathway in the Unfolded Protein Response

The IRE1 pathway is the most evolutionarily conserved branch of the UPR, and plays a crucial role in protecting cells from damage and cell death. Within the UPR signaling pathway, IRE1α is a key molecule. In mammals, there are two homologs of IRE1: IRE1α and IRE1β. IRE1α is widely expressed across various cell types and is a transmembrane protein featuring an N-terminal luminal sensor domain, a transmembrane domain, and a C-terminal cytoplasmic effector domain with crucial protein kinase and endonuclease activities. By contrast, IRE1β is predominantly localized to gastrointestinal epithelial cells and exhibits distinct structural features in its luminal domain that confer tissue-specific stress responsiveness [14].

The accumulation of unfolded proteins in the ER triggers the oligomerization and autophosphorylation of IRE1α, leading to the activation of its RNase domain. This RNase domain is responsible for splicing X-box binding protein 1 (XBP1) mRNA, resulting in the production of functional XBP1 protein. Additionally, IRE1α influences cell survival and stress responses by cleaving its own mRNA and regulating microRNAs [15,16,17,18]. IRE1β, on the other hand, is primarily found in intestinal epithelial cells. Both proteins are localized to the ER, where they transmit signals and activate downstream ER stress responses. XBP1s functions as a potent transcription factor that induces ER chaperones, thereby regulating the adaptive response of cells to ER stress. Cells lacking XBP1 are more susceptible to oxidative stress and inflammation, highlighting the significance of XBP1 in cellular protection and metabolism [19].

In addition to its role in XBP1 splicing, IRE1α mediates the cleavage of ER-targeted mRNAs, alleviating the burden of newly synthesized proteins on the ER. This process is known as regulated IRE1-dependent decay (RIDD). RIDD not only regulates the generation and degradation of microRNAs but also affects the expression of target proteins [20,21]. For instance, persistent activation of IRE1α leads to the cleavage of specific microRNAs, thereby enhancing the expression of the proapoptotic protein [22].

In summary, IRE1α plays multiple roles in the unfolded protein response, not only by regulating cellular stress responses through XBP1 but also by affecting mRNA and microRNA through RIDD. Future research will further elucidate the importance of IRE1α and its pathways in various physiological and pathological processes.

### 2.3. The Role of PERK in the Unfolded Protein Response

PERK (PKR-like ER kinase) is a serine/threonine protein kinase located on the ER membrane, primarily responsible for attenuating mRNA translation during ER stress. This mechanism effectively prevents the influx of newly synthesized proteins into an already stressed ER compartment. Upon activation by ER stress, PERK undergoes dimerization and autophosphorylation, which initiates a cascade of events that leads to the phosphorylation of eukaryotic translation initiation factor 2 (eIF2α) [23,24].

Phosphorylation of eIF2α inhibits the guanine nucleotide exchange factor eIF2B, which is essential for recycling the GTP-bound form of eIF2α necessary for the initiation of polypeptide synthesis. This attenuation of general protein translation serves to alleviate the burden on the ER; however, it also preferentially enhances the translation of specific UPR-related genes, notably activating transcription factor 4 (ATF4). The 5′ untranslated region of ATF4 mRNA contains an upstream open reading frame (uORF), allowing it to bypass the translation inhibition imposed by phosphorylated eIF2α [25,26].

In addition to its effects on eIF2α, PERK also phosphorylates nuclear factor erythroid 2-related factor 2 (NRF2), a key regulator of the antioxidant response [27]. This phosphorylation promotes the expression of genes containing antioxidant response elements (AREs), aiding in the cellular defense against oxidative stress. For instance, PERK-mediated activation of NRF2 leads to the upregulation of heme oxygenase-1 (HO-1) and other antioxidant genes, which helps ease oxidative damage during ER stress [28]. This circuitous relationship between ER stress and compensation through the oxidative stress reduction pathway is one of the first instances of ER-to-nuclear regulation of ER stress.

### 2.4. ATF6

During ER stress, ATF6 (activating transcription factor 6) is activated through a distinct mechanism compared to other ER stress sensors, such as IRE1 and PERK. Upon ER stress, ATF6 translocates to the Golgi apparatus [29], where it undergoes proteolytic cleavage. Once cleaved, ATF6 is transported to the nucleus, where it plays critical roles in ER-associated degradation (ERAD) and apoptosis [30,31].

In the nucleus, ATF6 can bind to XBP1 [32], enhancing the expression of XBP1 and other chaperones. One of the protective functions of ATF6 is its ability to induce the expression of protein disulfide isomerase associated 6 (PDIA6), an ER enzyme that catalyzes the formation of disulfide bonds, thereby facilitating protein folding within the ER lumen [33,34].

Recent studies have further confirmed the pivotal role of ATF6 in maintaining the functional integrity of the ER in stressed cells. On one hand, enhancing ATF6 activity can upregulate protective molecules, such as GRP78, which significantly improves myocardial injury and reduces apoptosis and necrosis in cardiac tissues subjected to ischemia/reperfusion (I/R) injury [35]. On the other hand, ATF6-mediated downregulation of miR-455 can enhance the expression of calreticulin, effectively alleviating ER stress following ischemic events [35].

In summary, ATF6 is a crucial player in the cellular response to ER stress, facilitating protective adaptations that help maintain ER function and promote cell survival. Understanding the mechanisms by which ATF6 operates may provide valuable insights for therapeutic strategies targeting conditions associated with ER stress and cellular dysfunction.

### 2.5. GPX8

Protein misfolding is a critical cellular challenge that occurs when proteins fail to achieve their proper three-dimensional conformation during synthesis or post-translational modifications [36]. This misfolding can result from various factors, including genetic mutations, environmental stressors (such as changes in temperature and pH), oxidative stress, and dysfunction of the endoplasmic reticulum (ER). Under normal circumstances, chaperones and enzymes assist in ensuring correct protein folding and maintaining cellular homeostasis. However, when these mechanisms are compromised, misfolded proteins can accumulate, leading to significant cellular consequences [37].

The consequences of misfolded proteins are multifaceted. They can impair cellular functions. Properly folded proteins are essential for a myriad of biological processes, including enzyme catalysis, signal transduction, and structural integrity. Misfolded proteins often lack functional capabilities, disrupting metabolic pathways and cellular signaling, which can result in abnormal cell behavior and contribute to various diseases such as Alzheimer’s disease, Parkinson’s disease, Huntington’s disease, Creutzfeldt–Jakob disease, cystic fibrosis, Gaucher’s disease, and many other degenerative and neurodegenerative disorders [3].

In this context, the role of GPX8 (glutathione peroxidase 8) in maintaining ER function and stability becomes increasingly relevant. GPX8 is an antioxidant enzyme that helps lessen oxidative stress by scavenging peroxides within the ER. By reducing peroxide levels, GPX8 plays a crucial role in protecting the ER from oxidative damage, which is essential for proper protein folding and function. The enzyme’s activity helps maintain the delicate balance of redox status within the ER, thereby supporting the function of chaperones and preventing the accumulation of misfolded proteins [38,39,40]. By protecting the ER from oxidative stress, GPX8 indirectly contributes to cellular resilience against conditions that typically lead to protein misfolding. Enhanced GPX8 activity can reduce the burden of reactive oxygen species (ROS), thereby preventing the cascade of events that leads to misfolding and aggregation. This protective role underscores the importance of antioxidant mechanisms in the ER, particularly in the context of stress responses. Moreover, Nguyen found that catalytically active members of the PDI family may serve as the natural partners of GPX8 in vivo [41], and further demonstrated their co-localization with endoplasmic reticulum oxidoreductase 1 (ERO1α), a process that facilitates functional communication. The relevance of GPX8 (glutathione peroxidase 8) in the detoxification of hydrogen peroxide (H_2_O_2_) produced by deregulated ERO1 (endoplasmic reticulum oxidoreduction 1) has been substantiated through various cellular studies. When ERO1 activity becomes dysregulated, it can lead to excessive H_2_O_2_ production, resulting in oxidative stress that compromises cellular function and protein integrity. In this context, GPX8 plays a pivotal role by scavenging H_2_O_2_, thereby mitigating the oxidative damage that can ensue from ERO1 overactivity. By converting H_2_O_2_ into water, GPX8 helps maintain the redox balance within the ER, which is essential for the proper folding of proteins. This detoxification process not only prevents the accumulation of misfolded proteins but also supports the activity of chaperones that assist in correct protein folding [42,43,44]. Ablation of GPX8 leads to the induction of ER stress and cell death owing to the outflow of H_2_O_2_ from the ER into the cytosol. Granatiero found that GPX8 also plays a vital role in ER oxidative protein folding, and H_2_O_2_ and calcium (Ca^2+^) homeostasis [45]. Interestingly, GPX8 overexpression lessens both Ca^2+^ storage in the ER and histamine-induced Ca^2+^ release, indicating a resistance to ferroptosis [46,47].

### 2.6. GPX8 and Disease

GPX8 expression may contribute to resistance against regulated cell death [48]. Bosello found that there is a link between GPX8 and epithelial–mesenchymal transition (EMT), which is associated with cancer metastasis [49]. Ren observed the role of GPX8 in cancer immunology, highlighting its potential implications in the immune response [50]. Furthermore, Khatib emphasized the indispensable role of GPX8 in maintaining an aggressive breast cancer phenotype, while Nguyen discovered that GPX8 is involved in renal cell carcinoma tumorigenesis, indicating that GPX8 could serve as a novel prognostic factor, therapeutic target, and marker of EMT across various cancer subtypes [51,52,53]. Furthermore, according to patient data from the Human Protein Atlas, GPX8 exhibits significant differential expression across all cancer types, particularly in glioma, lung cancer, and ovarian cancer (unfavorable), suggesting its potential role as a prognostic marker for these malignancies [54,55,56]. GPX8 plays a role in radiation treatment—knockdown of GPX8 in BxPC-3 pancreatic cancer cells enhances radiation sensitivity [57].

The loss of GPX8 has been shown to result in a reduction in membrane polyunsaturated fatty acids (PUFAs). This suggests that the absence of GPX8 may cause cells to retain higher levels of PUFAs. Consequently, GPX8 is implicated in lipid remodeling, which could play a role in the development of obesity [48,53,58,59,60].

Beyond cancer, GPX8 has also been implicated in immune regulation, viral pathogenesis, and metabolic diseases. It was shown that GPX8-deficient mice were more susceptible to colitis, and exhibited increased caspase-4/11 activation, with higher levels of caspase-induced inflammation during colitis and septic shock [61]. Proteomics studies in inducible expression cell lines of hepatitis C virus NS3-4A protease revealed that GPX8, a pro-viral host factor, is cleaved by NS3-4A protease at Cys11, removing the cytosolic tip of GPX8. This cleavage of GPX8 was also observed in liver biopsies from patients with chronic HCV [62]. In insulin-secreting INS-1E cells, GPX8 mitigates palmitate-induced ER Ca^2+^ depletion by detoxification of luminal H_2_O_2_ [63]. The expression of GPX7 or GPX8 attenuated saturated fatty acid-mediated H_2_O_2_ generation, ER stress, and apoptosis induction in INS-1E cells [43]. In general, the dual roles of GPX8 in ER redox homeostasis and disease have been highlighted in recent years (Table 1).

### 2.7. Plant GPXs Have Parallel Functions in ER Redox Stress and UPR

The plant UPR exhibits a distinct composition and regulatory mode compared to that of animals, such as the absence of the PERK pathway and a reliance on the bZIP17/28 and NAC transcription factors. Investigations of the conserved and divergent components in plant and animal UPR systems will provide novel insights into the functional mechanisms and evolutionary significance of different UPR regulatory modules. Of particular interest is whether plant GPXs play a significant role in the UPR system.

In plant systems, diverse biotic (e.g., pathogen invasion) and abiotic stressors (including drought and salinity) trigger oxidative imbalances within the ER, thus promoting the UPR. The immobility of most plants has selected robust ER stress mitigation strategies. Unlike animals, who employ avoidance behaviors, plants have evolved multilayered detoxification systems (peroxidases, chaperone networks, and selective autophagy) to counterbalance elevated ROS generation at ER membranes. Generally, plant peroxidases are classified into two main groups: heme-containing peroxidases (peroxidase–catalase superfamily) and non-heme peroxidases (thiol peroxidase superfamily) [64]. In recent decades, the importance of non-heme peroxidases has garnered research interest. Among these, the thiol peroxidase superfamily is well characterized and is primarily divided into peroxiredoxins (PRXs) and glutathione peroxidases (GPXs) [65].

GPXs evolved from a monomeric ancestor, with early divergence between animal and TRX-preferring fungal and plant GPXs [66]. All plant GPXs show high homology to the animal GPX4 cluster [67], and similar to animal GPX4, plant GPXs lack polymerization domains and typically do not function as oligomers, while other animal GPXs can form dimers or tetramers [68]. However, some reports indicate that GPXs in other plants can form dimers [69], suggesting different evolutionary strategies and functional differentiation between plant and animal GPXs.

In mammals, GPXs play crucial roles in the ER stress response and ferroptosis. Plant GPXs, unlike animal GPXs, bind TRX substrates with higher affinity than GSH substrates, consistent with a potential redox sensor role. The involvement of plant GPXs in ER oxidative stress and the UPR remains controversial; it was previously believed that other antioxidant enzymes primarily scavenge ROS in the ER. However, AtGPX3 has been shown to localize in the lumen and anchor to the ER and Golgi membranes [70,71], while transmembrane domains were also identified in other plant GPXs [72]. Interestingly, the ectopic expression of *Arabidopsis* GPXs in HeLa cells demonstrated that all eight AtGPXs exhibit partial localization in the plasma membrane and cytosol, except for AtGPX3 and AtGPX6, indicating that plant GPXs may also function within the membrane systems of mammalian cells [73].

The UPR in plants is orchestrated by a conserved signaling network primarily consisting of two branches: IRE1-bZIP60 and bZIP17/28, as noted in reference [74]. There is a paucity of studies in the literature specifically addressing the roles of GPXs within the plant UPR framework. However, it has been observed that treatment with the ER stress inducer tunicamycin leads to an increase in intracellular concentrations of oxidized glutathione and a reduction in the GSH/GSSG ratio, as highlighted in reference [75]. Furthermore, glutathione (GSH)-related signaling pathways appear to play a significant part in ER stress and UPR, as evidenced by the decreased upregulation of autophagy-related protein 8 (ATG8) following GSH treatment. This suggests an interconnection between GSH signaling and the UPR process in plants. Additionally, thioredoxin-related protein disulfide isomerases (PDIs) are involved in the folding of nascent and misfolded proteins within the secretory pathway, implying that TRX-catalyzed GPXs might have a role in this process, as indicated in reference [76]. Moreover, the loss of function of GPX members in plants has been associated with increased sensitivity to oxidative stress and subsequent activation of the UPR, emphasizing the importance of GPXs in maintaining cellular redox homeostasis and mitigating oxidative damage [77,78].

## 3. Discussion and Summary

The delicate balance between redox homeostasis and endoplasmic reticulum (ER) function is critical for cellular health and survival. ROS, while essential for signaling pathways, can become harmful when levels exceed the cellular antioxidant capacity, leading to oxidative stress and damage to proteins, lipids, and DNA. The ER, as a central hub for protein folding and post-translational modifications, is particularly vulnerable to oxidative stress, which can disrupt its function and trigger the UPR. The UPR serves to protect and restore ER homeostasis by enhancing protein folding capacity, reducing protein load, and promoting the degradation of misfolded proteins. However, prolonged or severe ER stress can lead to cell death, highlighting the importance of maintaining ER function and redox balance.

**Figure 1:**
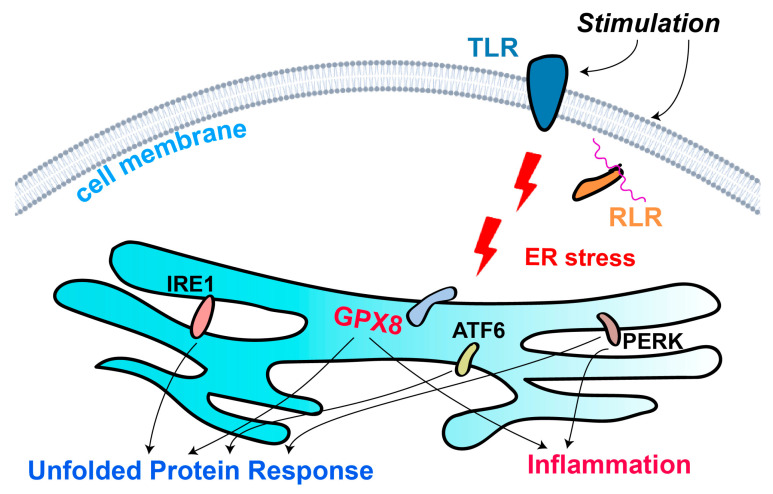
Schematic representation of GPX8’s role in ER redox homeostasis and UPR. This figure illustrates the role of GPX8 in maintaining ER redox homeostasis and its interaction with the UPR pathways. GPX8 detoxifies H_2_O_2_ produced during oxidative protein folding, preventing oxidative damage and supporting proper protein folding. The UPR pathways (IRE1α, PERK, and ATF6) are activated in response to ER stress, enhancing protein folding capacity and reducing the protein load. GPX8’s activity helps mitigate the oxidative stress associated with ER stress, thereby supporting cell survival and function.

GPX8 emerges as a key player in this context, acting as a critical antioxidant enzyme within the ER. By detoxifying H_2_O_2_ produced during oxidative protein folding, GPX8 helps maintain the redox balance necessary for proper protein folding and function. Its role in mitigating oxidative stress is particularly important in preventing the accumulation of misfolded proteins, which can lead to ER stress and contribute to various diseases, including neurodegenerative disorders and cancer. Furthermore, GPX8’s involvement in calcium homeostasis and its potential role in lipid remodeling underscore its multifaceted contributions to cellular health (Figure 1).

In cancer, GPX8 has been implicated in promoting tumor aggressiveness and metastasis, particularly through its association with EMT. This dual role of GPX8—both protective and potentially pathogenic—highlights the complexity of redox regulation in disease contexts. While GPX8 overexpression may confer resistance to cell death in cancer cells, its loss can sensitize cells to oxidative stress and ER stress, suggesting that targeting GPX8 could be a viable therapeutic strategy in certain cancers.

In plants, GPXs play a similarly crucial role in responding to oxidative and ER stress, although their mechanisms and substrates differ from those in animals. Plant GPXs, particularly those in the thiol peroxidase superfamily, are involved in maintaining redox homeostasis and mitigating the effects of biotic and abiotic stresses. The conservation of GPX function across kingdoms underscores the fundamental importance of redox regulation in cellular survival and adaptation to stress.

## 4. Conclusions

In particular, GPX8 plays a pivotal role in maintaining ER redox homeostasis and mitigating oxidative stress, which is essential for proper protein folding and cellular function. Its involvement in various physiological and pathological processes, including cancer and neurodegenerative diseases, highlights its potential as a therapeutic target. Further research into the mechanisms of GPX8 and its interactions with other cellular pathways will provide valuable insights into its role in health and disease.

## Figures and Tables

**Table 1:** Dual roles of GPX8 in ER redox homeostasis and disease.

Key Findings on GPX8	Dual Roles Highlighted	References
GPX8 localizes near ERO1α, detoxifying H_2_O_2_ produced during oxidative protein folding.	Redox balance in ER. Prevents H_2_O_2_ leakage to cytosol.	[41]
GPX8 regulates ER Ca^2^⁺ homeostasis and H_2_O_2_ scavenging; overexpression resists ferroptosis.	Antioxidant. Modulates Ca^2^⁺ signaling.	[45,46,47]
GPX8 links to EMT in cancer; ablation reduces membrane PUFAs, implicating lipid remodeling.	Pro-metastatic. Lipid metabolism regulator.	[49,53,58,59,60]
GPX8 influences cancer immunology, suggesting immune response modulation.	Tumor progression. Immune evasion.	[50]
GPX8 maintains aggressive breast cancer phenotype; prognostic marker in multiple cancers.	Pro-survival in cancer. EMT promoter.	[51,52,53,54]
GPX8 is cleaved by HCV NS3-4A protease (Cys11), disrupting ER redox defense in chronic HCV.	Antiviral host factor. Viral pathogenesis target.	[62]
GPX8 mitigates palmitate-induced ER Ca^2^⁺ depletion by detoxifying luminal H_2_O_2_.	Protects β-cells. Reduces ER stress in metabolic disease.	[43,63]

## Data Availability

The original contributions presented in this study are included in the article. Further inquiries can be directed to the corresponding author(s).
